# The burden and predictors of latent tuberculosis infection among elder adults in high epidemic rural area of tuberculosis in Zhejiang, China

**DOI:** 10.3389/fcimb.2022.990197

**Published:** 2022-10-27

**Authors:** Wei Wang, Xinyi Chen, Songhua Chen, Mingwu Zhang, Wei Wang, Xiaogang Hao, Kui Liu, Yu Zhang, Qian Wu, Ping Zhu, Bin Chen

**Affiliations:** ^1^ Department of Tuberculosis Control and Prevention, Zhejiang Provincial Center for Disease Control and Prevention, Hangzhou, Zhejiang, China; ^2^ Department of AIDS and Tuberculosis Control and Prevention, Quzhou Center for Disease Control and Prevention, Quzhou, Zhejiang, China

**Keywords:** tuberculosis, latent tuberculosis infection, risk factors, older adults, observational study

## Abstract

Diagnosis and treatment of latent tuberculosis infection (LTBI) is critical to tuberculosis (TB) control. Identifying the risk factors associated with LTBI can contribute to developing an optimized strategy for LTBI management. We conducted a survey of adults aged 65 years and older living in rural areas in Zhejiang Province during July 2021, followed by a one-year follow-up period to determine TB incidence. Participants underwent a physical examination and 5–6 mL of blood was drawn to test for Mycobacterium tuberculosis infection A total of 1856 individuals participated in the study, of whom 50.5% were men and 80.1% were married. Most participants (96.8%) often opened windows for ventilation at home. One-third (33.4%) of participants had abnormal chest radiographs and 34.9% had LTBI. Nine participants (0.5%) developed active TB patients during the one-year follow-up period. People who frequented closed entertainment places such as chess and card rooms had a relatively high percentage of LTBI (39.5%). Factors associated with a higher risk of LTBI in multivariable logistic regression analysis included being male (odds ratio [OR]:1.32; 95% confidence interval [CI] =:1.01-1.72), smoking (OR: 1.43; 95% CI:1.04-1.97), not opening windows for ventilation at home frequently (OR: 1.88; 95% CI: 1.10–3.22), and abnormal chest radiographs (OR; 1.48; 95% CI; 1.20–1.81). LTBI was prevalent among the elder adults living in high-epidemic rural areas of TB in Zhejiang province. Men, people who smoke, and people without the habit of ventilating at home should be targeted for LTBI screening to accelerate the decline of the TB epidemic in Zhejiang Province.

## Introduction

Tuberculosis (TB) is caused by the bacteria *Mycobacterium tuberculosis* (Mtb) and is responsible for 1.5 million deaths each year worldwide (World Health Organization, 2021). Mtb is often in a quiescent state in an infected person and does not cause disease quickly. The state in which people are latently infected with Mtb and without clinical evidence of active TB is referred to as latent tuberculosis infection (LTBI). The risk of LTBI developing into active TB could be 5%-10% over a lifetime (Andrews et al., 2012). About a quarter of the global population (approximately 2 billion individuals) have LTBI (World Health Organization, 2021), which is the precursor to TB disease and poses a great threat to TB control and the End TB Strategy, launched by the World Health Organization (WHO). The End TB Strategy aimed to achieve the ambitious goal of 90% and 95% reduction respectively in TB incidence and mortality by 2035 (Executive Board, 2014). Early diagnosis and treatment of LTBI might reduce the risk of developing TB disease and interrupt the spread of TB. Based on this, WHO advocates screening for LTBI and preventive treatment in high-risk groups (World Health Organization, 2018).

China is only one of the 30 countries with a high TB burden in the world and is also one with a high burden of LTBI (Gao et al., 2022). In order to reach the goal of the End TB Strategy, China is facing the challenge of developing a national strategy to address the further development of LTBI, including understanding the epidemiological characteristic of LTBI, identifying the target population for early screening and diagnosis, and development of preventive treatment regimens appropriate for the Chinese people (Xin et al., 2019). The fifth national TB prevalence survey conducted in China showed that TB prevalence increased with age (Technical Guidance Group of the Fifth National TB Epidemiological Survey, 2012). Studies found older adults were vulnerable to Mtb infection due to factors such as reduced immune function, complications, and malnutrition, (Donald et al., 2010; Pratt et al., 2011; Rajagopalan, 2016). Recently published articles showed the rate of LTBI in people aged 15 years and above was 20.3%, and the infection rate also increased with age (Gao et al., 2022). Several multicenter studies have reported that the burden of LTBI and annual Mtb infection rate were both significantly higher in older people compared with younger individuals in rural areas of China (Gao et al., 2015; Gao et al., 2016). Hence, intensifying efforts should be enhanced to prioritize LTBI detection and preventive treatment in high-risk groups, particularly in older adults. Modeling analysis also suggested that successful preventive therapy for the elderly population would reduce the incidence of TB in China by 84% (Huynh et al., 2015).

In China, about 71% of patients with TB live in rural areas (Wang et al., 2007; Technical Guidance Group of the Fifth National TB Epidemiological Survey, 2012; Wang et al., 2014). Therefore, rural must be the priority areas where a lot more investment and effective screening tools are needed to reduce the reservoir of potential TB cases. Zhejiang Province, located in southeastern China, has a relatively well-developed economy and a middle-level notification rate of TB in China (Ge et al., 2016). However, the western regions of the province are located in a mountainous area and still suffer from a high risk of TB. The burden of LTBI in these areas was unknown. This study aimed to identify the prevalence of and factors associated with LTBI in rural areas with a high TB incidence, which might contribute to optimizing intervention strategy for LTBI in rural areas of China, and provide a scientific basis for the development of TB prevention and control strategies.

## Materials and methods

### Study design and participants

We conducted an observational study in rural areas in Zhejiang Province. Two counties were selected in rural areas with high incidences of tuberculosis in Zhejiang Province. In each county, the township with the highest TB prevalence in the five years of 2016-2020 was selected. Three villages were randomly selected in each selected township. In selected villages, all eligible subjects were surveyed. Inclusion criteria: a. Local resident or permanent resident with at least 6 months of continuous residence in the latest year; b. Aged 65 or older at the time of the survey; c. Voluntarily participate in this study and sign informed consent. Exclusion criteria: a. Tuberculosis patient; b. History of tuberculosis; c. Unwilling to participate in the study. Only those who meet all inclusion criteria and none of the exclusion criteria could be included in the study. The baseline cross-sectional survey was conducted in July 2021, followed by one year of surveillance.

The sample size was calculated based on the following formula (Bacchetti et al., 2005),


n=(Zα2)2p(1−p)δ2

in which n is the required sample size in one research county and *p* is the estimated prevalence of LTBI. δ is allowed a margin of error. The value of *p* was estimated to be 30% according to a study in China (Gao et al., 2015). After adding a 10.0% error for the study, a total of 354 individuals in each county site were needed for the analysis.

### Data collection

According to the Basic Public Health Service Standards of Zhejiang Province (4th edition) (Zhejiang Provincial Health and Family Commission, 2017), the community health service institutions should provide physical health examinations for local residents aged 65 and above annually. The study was performed combined with the residents’ health physical examination by trained and qualified medical personnel and investigators. During the health physical examination, the basic demographic characteristics and social behavior which included sex, age, education, marriage, smoking status, alcohol consumption, and frequently open windows for ventilation at home were asked by trained investigators.

People who smoke was defined as an individual who smoked at least 1 cigarette per day for more than 6 months in the past year. Participants who had formerly smoked were defined as smokers who had been smoke-free for at least 6 months at the time of the survey. Alcohol consumption was defined as having consumed alcohol in the past 12 months and consuming more than 20 mL of alcohol. Daily alcohol consumption was defined as consuming at least 20 mL of alcohol per day. Frequent alcohol consumption was defined as consuming more than 20 mL of alcohol on average 1–3 times per week. Occasionally alcohol consumption is defined as drinking on average 1–3 times per month and consuming more than 20 mL of alcohol. Opening windows frequently for ventilation was defined as ventilating the family space or bedroom at least three times per week for at least 1 hour at a time. Places of frequent activity were defined as public places in which participants spent no less than 1 hour per time and at least 2 times a week.

The height, weight, and chest radiographs were conducted by medical personnel. In addition, about 5-6mL of blood from each participant was drawn to test for Mtb infection using IFN-γ release assays by professional medical staff. Participants in one county were tested for Mtb infection using T-SPOT.TB (CFDA20183400233) and participants in another county were tested with QuantiFERON-TB Gold (CFDA(I)20133405272). Both the methods had been registered and certified by the China State Food and Drug Administration, which could be used for auxiliary diagnosis of clinical tuberculosis and specific detection of tuberculosis infection caused by Mtb. Laboratory tests were carried out in strict accordance with the reagent instructions. Quality control of the laboratory testing process was carried out by the provincial laboratory workers. A normal chest radiograph was defined as having no abnormal lesions. An abnormal chest radiograph was defined as the presence of partial lesions, in which the possibility of active pulmonary TB was excluded by a radiographer. The state of people latently infected with Mtb but without clinical evidence of active TB is to be referred to as LTBI. The diagnosis of LTBI was also carried out by professional community health service personnel.

### Statistical analysis

Descriptive statistics were used to describe the participants’ characteristics. A Chi-square test was conducted to detect the association between LTBI and participants’ characteristics. Variables with a p-value less than 0.1 in the chi-square tests were included in the multivariable analysis. Factors associated with LTBI were analyzed using multivariable logistic regressions, with the estimation of their odds ratios (ORs) and 95% confidence intervals (CIs). The “Enter” method was used in logistic regressions. The cumulative incidence risk of participants with LTBI positive compared to LTBI negative participants was also explored by multivariable logistic regressions when adjusted for sex, age, education, marital status, BMI, smoking, drinking, the habit of opening windows often for ventilation, and the chest radiograph result. A P value less than 0.05 was considered statistically significant. All statistical analysis was conducted using SPSS version 19.0 (IBM Corp, Armonk, NY, USA).

## Results

### Social-demographic characteristics of participants

Of the 2054 people aged 65 or older in the 6 selected villages, 1903 consented to participate in this study; 47 individuals were excluded due to active TB disease or a history of active TB. Finally, 1856 (90.4%) individuals were enrolled in the study.

The characteristics of all the 1856 participants included in the study are shown in **Table 1**. Approximately half of the participants were men (50.5%). The age ranged from 65 to 99 years with a mean age of 72.86 years. About 48.5% of participants had a primary school education level, and 38.1% were illiterate or semiliterate. More than two-thirds of participants reported they had never smoked (70.0%) and never drank alcohol (74.8%). Most participants (96.8%) frequently opened windows for ventilation at home. About two-thirds of participants had normal chest radiographs, and 33.4% of participants had abnormal chest radiographs. Among all the participants, 34.9% were diagnosed with LTBI.

**Table 1:** Characteristics of study participants.

Items	n	%
**Sex**		
Female	938	50.5
Male	918	49.5
**Age (years)**		
65-70	661	35.6
70-75	591	31.8
75-80	317	17.1
80-	287	15.5
**Education**		
Illiterate and semiliterate	708	38.1
Primary school	900	48.5
Junior high school and above	248	13.4
**Marriage**		
Married	1487	80.1
Unmarried	46	2.5
Widowed/divorced	323	17.4
**BMI**		
<18.5	179	9.9
18.5-24	1063	58.8
24-28	449	24.8
28-	116	6.4
**Smoking status**		
People who never smoked	1283	70.9
People who smoke currently	336	18.6
People who formerly smoked	190	10.5
**Alcohol consumption**		
Never	1384	74.8
Occasionally	105	5.8
Frequently	22	1.2
Daily	328	18.1
**Open Windows frequently for ventilation at home**		
Yes	1794	96.8
No	59	3.2
**Chest radiograph results**		
Normal	1238	66.7
Abnormal	618	34.3
**LTBI results**		
Negative	1199	64.1
Positive	644	34.9
**Developed active TB during the one-year follow-up period**		
No	1844	99.5
Yes	9	0.5

LBTI, latent tuberculosis infection; TB, tuberculosis; BMI, Body Mass Index.

The total is less than 1856 due to missing values.

### Prevalence of LTBI according to participant characteristics

**Table 2** shows the association between participant characteristics and LTBI. No statistically significant differences were observed in different age groups, education levels, marriage status, and body mass index (BMI). The chi-square test found that 5 variables were associated with LTBI infection: sex, cigarette consumption, alcohol consumption, the habit of opening windows frequently for ventilation or not, and chest radiographs. The percentage that were LTBI positive in men was higher than in women (40.6% versus 29.4%), the difference between the 2 groups was significant (p<0.01). More percent of people who smoke (45.5%) were LTBI positive than participants who never smoke (31.7%) and formerly smoked (39.7%). The proportion of LTBI positive in participants with alcohol consumption was higher than in participants without alcohol consumption. About 48.3% of participants who did not often open windows for ventilation at home, were LTBI positive, which was higher than that of participants who often opened windows for ventilation at home (34.5%). Participants with abnormal chest radiographs had a higher LTBI positive proportion than participants with normal chest radiographs (41.0% versus 31.9%, p<0.001). People who frequented closed entertainment places such as chess and card rooms had a relatively high prevalence of LTBI (39.5%).

**Table 2:** The relationships between participant characteristics and latent tuberculosis infection in the study.

Items	Latent tuberculosis infection					
	Negative		Positive			
	n	%	m	%	χ²	P
**Age**					**2.91**	**0.406**
65-70	438	66.7	219	33.3		
70-75	379	64.2	211	35.8		
75-80	207	66.6	104	33.4		
80-	175	61.4	110	38.6		
**Sex**					**25.79**	**<0.001**
Female	657	70.6	273	29.4		
Male	542	59.4	371	40.6		
**Education**					**3.89**	**0.143**
Illiterate and semiliterate	471	67.2	230	32.8		
Primary school	579	64.7	316	35.3		
Junior high school and above	149	60.3	98	39.7		
**Marriage**					**4.21**	**0.122**
Married	966	65.1	517	34.9		
Unmarried	23	51.1	22	48.9		
Widowed/divorced	210	66.7	105	33.3		
**BMI**					**5.932**	**0.155**
<18.5	126	71.6	50	28.4		
18.5-24	668	63.3	388	36.7		
24-28	301	67.2	147	32.8		
28-	73	62.9	43	37.1		
**Smoking status**					**24.47**	**<0.001**
People who never smoke	872	68.4	403	31.6		
People who smoke	182	54.5	152	45.5		
People who formerly smoked	114	60.3	75	39.7		
**Alcohol consumption**					**14.86**	**0.02**
Never	904	67.3	439	32.7		
Occasionally	59	56.2	46	43.8		
Frequently	10	45.5	12	54.5		
Everyday	195	59.5	133	40.5		
**Open Windows frequently for ventilation at home**					**4.68**	**0.030**
Yes	1167	65.5	615	34.5		
No	30	51.7	28	48.3		
**Chest radiograph results**					**14.78**	**<0.001**
Normal	836	68.1	392	31.9		
Abnormal	363	59.0	252	41.0		
**Places of frequent activity (Multiple)**						
Chess and card room	181	60.5	118	39.5	3.24	0.07
The cultural hall	33	62.3	20	37.7	0.29	0.66
Corner shop	96	66.7	48	33.3	0.27	0.68
Nearby vegetable market	240	64.5	132	35.5	0.06	0.8
Neighborhood	237	66.9	117	33.1	0.68	0.4
Outdoor activity plaza	429	65.5	226	34.5	0.08	0.78
Seldom go out	512	64.5	282	35.5	0.21	0.64

The total is less than 1856 due to missing values.

### Factors associated with latent tuberculosis infection

Five characteristics were found to be associated with LTBI in the multivariate mode, as shown in **Table 3**. Male had a greater chance of LTBI infection than Female (OR: 1.32; 95% CI: 1.01–1.72). Participants who smoke had a greater chance of LTBI-positive infection than participants who never smoke (OR: 1.43; 95% CI: 1.04–1.97). Participants who did not often open windows for ventilation at home had a greater risk of TB infection than participants who often opened windows for ventilation at home (OR: 1.88; 95% CI: 1.10–3.22). Participants with abnormal chest radiographs had a greater chance of TB infection than participants with normal chest radiographs (OR: 1.48; 95% CI:1.20–1.81).

**Table 3:** Factors associated with latent tuberculosis infection among the elderly in rural area, Zhejiang, China.

		Latent tuberculosis infection	
Items	Positive/All Participants	P	OR (95%CI)
**Sex**			
Female	276/904		1
Male	375/891	0.043	1.32 (1.01-1.72)
**Smoking status**			
People who never smoke	419/1273		1
People who smoke	154/334	0.03	1.43 (1.04-1.97)
People who formerly smoked	78/188	0.70	1.08 (0.74-1.57)
**Alcohol consumption**			
Never	458/1341		1
Occasionally	48/104	0.181	1.34 (0.87-2.07)
Frequently	12/22	0.175	1.83 (0.77-4.36)
Everyday	133/328	0.957	1.01 (0.75-1.36)
**Open Windows frequently for ventilation at home**			
Yes	622/1738		1
No	29/57	0.022	1.88 (1.10-3.22)
**Chest radiograph results**			
Normal	378/1180		1
Abnormal	273/615	<0.001	1.48 (1.20-1.81)

The total is less than 1856 due to missing values.

### Annual incidence of tuberculosis among study participants

Of all the participants, 0.5% developed active TB cases during the one-year follow-up period. As shown in **Table 4**, the multivariable logistic regressions indicated the cumulative incidence rate of participants with LTBI positive was higher than that of LTBI negative participants (p<0.001). During the one-year observation period, the cumulative risk of developing TB disease in LTBI participants was nine times that of non-infected participants.

**Table 4:** The risk of tuberculosis incidence of participants during the one-year follow-up period according to baseline latent tuberculosis infection status.

	The cumulative tuberculosis incidence of participant during the one-year observation period				
	No TB (%)	TB (%)	Incidence Rate (per 100,000)	OR (95%CI)	AOR (95%CI)
LTBI results					
Negative	1197 (99.9%)	1 (0.1%)	100	1	1
Positive	635 (98.8%)	8 (1.2%)	1200	15.08 (1.88-120.84)	9.59 (1.07-86.20)

OR, Odds Ratio; AOR, Adjusted Odds Ratio; CI, confidence interval; LBTI, latent tuberculosis infection; TB, tuberculosis; The AOR was adjusted for sex, age, education, marital status, body mass index, smoking status, alcohol consumption, habit of opening windows frequently for ventilation, and the chest radiograph result.

The total is less than 1856 due to missing values.

## Discussion

Few studies have been conducted to estimate the burden of LTBI in older adults living in rural areas and explore the potential target for LTBI control in high epidemic areas of TB in Zhejiang province, China. This study showed that there was a high prevalence (34.9%) of LTBI among older adults living in rural areas, which was in line with the findings of studies in Jiangsu (31.2-33.33%) (Liu et al., 2017), but was lower than the estimation of the national burden of LTBI among people aged 60 years and older based a multi-center epidemiological survey in China (38.36%) (Gao et al., 2022).

The high prevalence of LTBI is a great threat to TB prevention and control. Studies showed that people with LTBI have a relatively high risk of developing active TB, especially within 5 years of the initial infection (Andrews et al., 2012). Our study also found a higher cumulative incidence rate in participants with LTBI positive than that of LTBI negative participants during our one-year follow-up period. Studies indicated that implementation of active case finding for LTBIs and tuberculosis preventive treatments nationwide or in areas with high incidence and in older adults could greatly reduce the number of TB cases, which may contribute to achieving the target of the “End TB Strategy” in China (Huynh et al., 2015; Wen et al., 2022). The WHO developed a series of recommendations for screening LTBI in developed countries with low incidence levels of active TB (Godoy, 2021). A study in Australia demonstrated that screening and management of LTBI patients can be achieved within the primary care setting, considering barriers and enablers at the patient, provider, and clinical levels (Kunin et al., 2022). The formulation of tuberculosis prevention and control strategies in rural areas with a high TB incidence should be tailored to local conditions, including available public health resources. China had established a relatively complete primary care system. Most community health service institutions could provide health physical examinations for the local residents annually, which made it possible to conduct screening and management of participants with LTBI in these primary care settings.

Although the incidence of TB had declined in recent years with the implementation of the DOTS strategy (Wang et al., 2007; Wang et al., 2014), about 800 thousand TB cases were still reported annually in China (World Health Organization, 2021). To reach the “End TB Strategy”, more efforts should be intensified in TB control, particularly in how LTBI intervention is effectively conducted to achieve a rapid decline in TB incidence in some important areas. An accurate understanding of the LTBI burden and epidemic characteristics in these areas is conducive to identifying the intervention targets and developing appropriate intervention technical guidelines for LTBI. Age was found to be associated with LTBI in previous studies. A study showed age older than 50 years exhibits a higher incidence of LTBI than younger ages in Taiwan (Chang et al., 2022). The rate of Mtb infection were found to gradually increase with age. Older people were more likely to acquire Mtb infection, but no statistically significant difference was observed in participants aged 60 years and older (Gao et al., 2015), which was similar with our study results. The effect of age on LTBI was also seen in Brazil (De Jezus et al., 2021) and Japan (Ogawa et al., 2021). Compared to the younger in Zhejiang, not only the prevalence of LTBI but also the incidence of tuberculosis was high in older adults (Zhu et al., 2021), which may be the result of the higher cumulative exposure risk and poor immunity with age increasing. Our study found male had a greater chance of TB infection than female, which was also supported in other countries (Fernandes et al., 2018; Lwin et al., 2020). The common interpretation was that men generally moved more widely and performed more physical types of employment, which may lead to a higher risk of TB exposure. A significant association between current cigarette consumption and risk of LTBI was observed in our study, which was also reported in other populations (Gao et al., 2015). Research has shown that cigarette consumption could suppress the protective immune response to Mtb (Shang et al., 2011), which may increase susceptibility to TB. A study in Colombia also showed that female sex and current cigarette smoking were associated with an increased risk of developing active TB (Herrera et al., 2021). Thus, screening for LTBI would be more effective in TB control among men, people who smoke, and older adults.

Participants who did not have the healthy habit of frequently opening windows for ventilation at home had a greater risk of Mtb infection than participants who had the healthy habit. It is common scientific knowledge that Mtb is carried in airborne particles and can spread through the air. Factors that determined the probability of transmission of Mtb included susceptibility, infectiousness, environment, and exposure. Environmental factors such as space, ventilation, air circulation, and air pressure could increase the risk of Mtb transmission. For example, inadequate local or general ventilation results in insufficient dilution or removal of infectious droplet nuclei (US Centers for Disease Control and Prevention, 2017). This could also explain the results that people who frequented closed entertainment places such as chess and card rooms had a relatively high percentage of LTBI positive. Therefore, keeping the environment ventilated is one of the measures to reduce the risk of Mtb transmission.

In addition to the screening for LTBI, the chest radiograph was also performed in order to exclude active disease or to reveal signs of old tuberculosis infection TB cases. Our study results showed the percentage of LTBI among participants with abnormal chest radiographs was higher than that of participants with normal chest radiographs. Similar results were also reported that lesions on chest radiographs suggestive of previous infection with Mtb were significantly associated with positive tests for LTBI (Uzorka et al., 2019). The complementary roles between chest radiograph and immunological testing methods were also reported (Wang et al., 2020). Thus, people with abnormal chest radiographs should also be the focus of screening for TB infection. It could improve the effectiveness of screening for LTBI to combine immunological testing methods and chest radiographs.

Our study has several limitations. Firstly, the study was based on the cross-sectional baseline study, which just demonstrated the association between different factors with LTBI. Secondly, there were differences in the detection efficacy of Mtb infection between the two methods (T-SPOT.TB and QuantiFERON-TB Gold) used in our study, but both the two methods had been certified by China State Food and Drug Administration, which could be used for specific detection of Mtb infection. Thirdly, the follow-up period was only one year, which was a little short, the incidence might vary with the length of follow-up. The initial infection time of the study subjects is unknown and different, which might also have an influence on morbidity. Despite the above deficiencies, our survey results can basically reflect the level of LTBI among older adults in areas of Zhejiang Province with a high TB incidence.

There was a high prevalence of LTBI among the older adults living in rural areas with a high burden of TB in Zhejiang province. Men, people who smoke and participants without the habit of ventilating at home were important targets for LTBI screening. Providing preventive intervention to people with LTBI would contribute to accelerating the decline of the TB epidemic.

## Data availability statement

The original contributions presented in the study are included in the article/supplementary material. Further inquiries can be directed to the corresponding authors.

## Ethics statement

The studies involving human participants were reviewed and approved by the Ethics Committee of the Zhejiang Provincial Center for diseases control and prevention (2021-027-01). The patients/participants provided their written informed consent to participate in this study.

## Author contributions

WW (1st author), PZ, and BC designed the study. WW (1st author) and XC wrote the manuscript. WW (1st author), BC, and SC modified the manuscript. WW (1st author), BC, and MZ did the statistics. XH, KL, QW, and YZ performed the investigation and collected data. WW (1st author), SC, MZ, and WW (5th author) were responsible for quality control of baseline investigation at the study sites. All authors contributed to the article and approved the submitted version.

## Funding

This study was funded by the foundation of the Medical and Health Science and Technology Project of Zhejiang (No.2020KY095) and the Quzhou Science and technology project(No. 2021K11).

## Acknowledgments

We thank all the medical workers and investigators from the study sites for their contribution to the study. We would like to thank the workers from Changshan County and Jiangshan Country Centers for Diseases Control and Prevention for their support with the field investigation.

## Conflict of interest

The authors declare that the research was conducted in the absence of any commercial or financial relationships that could be construed as a potential conflict of interest.

## Publisher’s note

All claims expressed in this article are solely those of the authors and do not necessarily represent those of their affiliated organizations, or those of the publisher, the editors and the reviewers. Any product that may be evaluated in this article, or claim that may be made by its manufacturer, is not guaranteed or endorsed by the publisher.

## References

[B1] Andrews J. R. Noubary F. Walensky R. P. Cerda R. Losina E. Horsburgh C. R. (2012). Risk of progression to active tuberculosis following reinfection with mycobacterium tuberculosis. Clin. Infect. Dis. 54 (6), 784–791. doi: 10.1093/cid/cir951 22267721 PMC3284215

[B2] Bacchetti P. Wolf L. E. Segal M. R. McCulloch C. E. (2005). Ethics and sample size. Am. J. Epidemiol. 161 (2), 105–110. doi: 10.1093/aje/kwi014 15632258

[B3] Chang A. Wu C. Z. Lin J. D. Lee C. N. Tsai K. Y. Wu P. H.. (2022). Prevalence and risk factors for latent tuberculosis among diabetes patients in Taiwan: A cross-sectional study. J. Infect. Dev. Ctries. 16 (4), 644–649. doi: 10.3855/jidc.15839 35544626

[B4] De Jezus S. V. do Prado T. N. Arcêncio R. A. Mascarello K. C. Sales C. M. M. Fauth M. M.. (2021). Factors associated with latent tuberculosis among international migrants in Brazil: A cross-sectional study, (2020). BMC Infect. Dis. 21 (1), 512. doi: 10.1186/s12879-021-06227-z 34074249 PMC8168318

[B5] Donald P. R. Marais B. J. Barry C. E. 3rd (2010). Age and the epidemiology and pathogenesis of tuberculosis. Lancet 375(9729), 1852–1854. doi: 10.1016/S0140-6736(10)60580-6 20488519

[B6] Executive Board (2014) Global strategy and targets for tuberculosis prevention, care and control after 2015 (World Health Assembly Secretariat). Available at: https://apps.who.int/iris/handle/10665/172828 (Accessed June 28 2022).

[B7] Fernandes P. Ma Y. Gaeddert M. Tsacogianis T. Marques-Rodrigues P. Fregona G.. (2018). Sex and age differences in mycobacterium tuberculosis infection in Brazil. Epidemiol. Infect. 146 (12), 1503–1510. doi: 10.1017/S0950268818001450 29880067 PMC6092217

[B8] Gao L. Bai L. Liu J. Lu W. Wang X. Li X.. (2016). Annual risk of tuberculosis infection in rural China: A population-based prospective study. Eur. Respir. J. 48 (1), 168–178. doi: 10.1183/13993003.00235-2016 27230438

[B9] Gao L. Lu W. Bai L. Wang X. Xu J. Catanzaro A.. (2015). Latent tuberculosis infection in rural China: Baseline results of a population-based, multicentre, prospective cohort study. Lancet Infect. Dis. 15 (3), 310–319. doi: 10.1016/S1473-3099(14)71085-0 25681063

[B10] Gao L. Zhang. H. Hu M. (2022). Expert consensus on the estimation of the national burden on latent tuberculosis infection. Chin J Antituberc. 44 (1), 4–8. doi: 10.19982/j.issn.1000-6221.20210662

[B11] Ge E. Zhang X. Wang X. Wei X. (2016). Spatial and temporal analysis of tuberculosis in Zhejiang Province, China 2009-2012. Infect. Dis. Poverty 5, 11. doi: 10.1186/s40249-016-0104-2 26906041 PMC4763446

[B12] Godoy P. (2021). Guidelines on controlling latent tuberculosis infection to support tuberculosis elimination. Rev. Esp. Sanid. Penit. 23 (1), 28–36. doi: 10.18176/resp.00028 33847703 PMC8278168

[B13] Herrera M. Keynan Y. López L. Marín D. Arroyave L. Arbeláez M. P.. (2021). Incidence and risk factors associated with latent tuberculosis infection and pulmonary tuberculosis among people deprived of liberty in Colombian prisons. Am. J. Trop. Med. Hyg. 106 (1), 66–74. doi: 10.4269/ajtmh.20-0307 34872056 PMC8733511

[B14] Huynh G. H. Klein D. J. Chin D. P. Wagner B. G. Eckhoff P. A. Liu R.. (2015). Tuberculosis control strategies to reach the 2035 global targets in China: The role of changing demographics and reactivation disease. BMC Med. 13, 88. doi: 10.1186/s12916-015-0341-4 25896465 PMC4424583

[B15] Kunin M. Timlin M. Lemoh C. Sheffield D. A. Russo A. Hazara S.. (2022). Improving screening and management of latent tuberculosis infection: Development and evaluation of latent tuberculosis infection primary care model. BMC Infect. Dis. 22 (1), 49. doi: 10.1186/s12879-021-06925-8 35022023 PMC8756639

[B16] Liu Y. Huang S. Jiang H. Xiong J. Wang Y. Ou M.. (2017). The prevalence of latent tuberculosis infection in rural jiangsu, China. Public Health 146, 39–45. doi: 10.1016/j.puhe.2017.01.008 28404472

[B17] Lwin T. T. Apidechkul T. Saising J. Upala P. Tamornpark R. Chomchoei C.. (2020). Prevalence and determinants of TB infection in a rural population in northeastern Myanmar. BMC Infect. Dis. 20 (1), 904. doi: 10.1186/s12879-020-05646-8 33256645 PMC7706037

[B18] Ogawa Y. Harada M. Hashimoto K. Kamijo Y. (2021). Prevalence of latent tuberculosis infection and its risk factors in Japanese hemodialysis patients. Clin. Exp. Nephrol. 25 (11), 1255–1265. doi: 10.1007/s10157-021-02093-w 34129132

[B19] Pratt R. H. Winston C. A. Kammerer J. S. Armstrong L. R. (2011). Tuberculosis in older adults in the united states 1993-2008. J. Am. Geriatr. Soc. 59 (5), 851–857. doi: 10.1111/j.1532-5415.2011.03369.x 21517786

[B20] Rajagopalan S. (2016). Tuberculosis in older adults. Clin. Geriatr. Med. 32 (3), 479–491. doi: 10.1016/j.cger.2016.02.006 27394018

[B21] Shang S. Ordway D. Henao-Tamayo M. Bai X. Oberley-Deegan R. Shanley C.. (2011). Cigarette smoke increases susceptibility to tuberculosis–evidence from *in vivo* and *in vitro* models. J. Infect. Dis. 203 (9), 1240–1248. doi: 10.1093/infdis/jir009 21357942

[B22] Technical Guidance Group of the Fifth National TB Epidemiological Survey (2012). The fifth national tuberculosis epidemiological survey in 2010. Chin. J. Antituberc. 34 (8), 485–508.

[B23] US Centers for Disease Control and Prevention (2017) Transmission and pathogenesis of tuberculosis. Available at: https://www.cdc.gov/tb/education/corecurr/pdf/chapter2.pdf (Accessed June 29 2022).

[B24] Uzorka J. W. Wallinga J. Kroft L. J. M. Ottenhoff T. H. M. Arend S. M. (2019). Radiological signs of latent tuberculosis on chest radiography: A systematic review and meta-analysis. Open Forum Infect. Dis. 6 (7) 1–7. doi: 10.1093/ofid/ofz313 PMC6667719 31363778

[B25] Wang P. H. Lin C. H. Chang T. H. Wu C. S. (2020). Chest roentgenography is complementary to interferon-gamma release assay in latent tuberculosis infection screening of rheumatic patients. BMC Pulm. Med. 20 (1), 232. doi: 10.1186/s12890-020-01274-9 32867745 PMC7461250

[B26] Wang L. Liu J. Chin D. P. (2007). Progress in tuberculosis control and the evolving public-health system in China. Lancet 369 (9562), 691–696. doi: 10.1016/S0140-6736(07)60316-X 17321314 PMC7134616

[B27] Wang L. Zhang H. Ruan Y. Chin D. P. Xia Y. Cheng S.. (2014). Tuberculosis prevalence in china 1990-2010; a longitudinal analysis of national survey data. Lancet 383 (9934), 2057–2064. doi: 10.1016/S0140-6736(13)62639-2 24650955

[B28] Wen Z. Li T. Zhu W. Chen W. Zhang H. Wang W. (2022). Effect of different interventions for latent tuberculosis infections in China: A model-based study. BMC Infect. Dis. 22 (1), 488. doi: 10.1186/s12879-022-07465-5 35606696 PMC9125978

[B29] World Health Organization (2018). Latent tuberculosis infection: Updated and consolidated guidelines for programmatic management. Licence: CC BY-NC-SA 3.0 IGO. 30277688

[B30] World Health Organization (2021). Global tuberculosis report 2021. Geneva, Licence: CC BY-NC-SA 3.0 IGO.

[B31] Xin H. Zhang H. Liu J. Pan S. Li X. Cao X.. (2019). Mycobacterium tuberculosis infection among the elderly in 20 486 rural residents aged 50-70 years in zhongmu county, China. Clin. Microbiol. Infect. 25 (9), 1120–1126. doi: 10.1016/j.cmi.2019.01.021 30738995

[B32] Zhejiang Public Health and Family Planning Commission (2017). The basic public health service standards of Zhejiang Province, 4th edition. (Zhejiang Province: Zhejiang Public Health and Family Planning Commission).

[B33] Zhu W. Wang Y. Li T. Chen W. Wang W. (2021). Gap to end-TB targets in eastern China: A joinpoint analysis from population-based notification data in Zhejiang Province, China 2005-2018. Int. J. Infect. Dis. 104, 407–414. doi: 10.1016/j.ijid.2021.01.007 33434670

